# Facilitators and barriers to home blood pressure monitoring among pregnant women in Ghana: a mixed-methods analysis of patient perspectives

**DOI:** 10.1186/s12884-024-06421-2

**Published:** 2024-03-19

**Authors:** Noah Newman, Titus K. Beyuo, Betty A. Nartey, Elorm Segbedzi-Rich, Andrea Pangori, Cheryl A. Moyer, Jody R. Lori, Samuel A. Oppong, Emma R. Lawrence

**Affiliations:** 1 University of Michigan Medical School, 1301 Catherine St., Ann Arbor, MI 48109 USA; 2 University of Ghana Medical School, P.O. Box 4236, Korle Bu, Accra, Ghana; 3 Korle Bu Teaching Hospital, Department of Obstetrics and Gynecology, University of Ghana Medical School, P.O. Box KB 77, Korle Bu, Accra, Ghana; 4 Department of Obstetrics and Gynecology, University of Michigan, 1500 E. Medical Center Dr., Ann Arbor, MI 48109 USA; 5 Department of Learning Health Sciences, University of Michigan, 1111 E. Catherine Street, Ann Arbor, MI 48109 USA; 6 University of Michigan School of Nursing, 400 N Ingalls St, Ann Arbor, MI 48104 USA

**Keywords:** Home blood pressure monitoring, HBPM, Preeclampsia, Eclampsia, Hypertensive disorder, Pregnancy, Ghana, LMIC

## Abstract

**Background:**

The benefit of home blood pressure monitoring during pregnancy and in low-resource settings is incompletely understood. The objective of this study was to explore the experiences, barriers, and facilitators of home blood pressure monitoring among pregnant women in Ghana.

**Methods:**

This concurrent triangulation mixed-methods study was conducted at an urban tertiary hospital in Ghana. Participants were recruited from adult pregnant women presenting for routine antenatal care. Upon enrollment, participants’ demographics and history were collected. At the next study visit, participants received audiovisual and hands-on training on using an automatic blood pressure monitor; they then monitored and logged their blood pressure daily at home for 2–4 weeks. At the final study visit, verbally administered surveys and semi-structured interviews assessed participant’s experiences. Quantitative data were analyzed using R version 4.2.2, and frequencies and descriptive statistics were calculated. Qualitative data were imported into DeDoose 9.0.78 for thematic analysis.

**Results:**

Of 235 enrolled participants, 194 completed surveys; of those, 33 completed in-depth interviews. Participants’ mean age was 31.6 (SD 5.3) years, 32.1% had not previously given birth, and 31.1% had less than a senior high school education. On a 4-point Likert scale, the majority reported they “definitely” were able to remember (*n* = 134, 69.1%), could find the time (*n* = 124, 63.9%), had the energy (*n* = 157, 80.9%), could use the blood pressure monitor without problems (*n* = 155, 79.9%), and had family approval (*n* = 182, 96.3%) while engaging in home blood pressure monitoring. 95.88% (*n* = 186) believed that pregnant women in Ghana should monitor their blood pressure at home. Qualitative thematic analysis demonstrated that most participants liked home blood pressure monitoring because of increased knowledge of their health during pregnancy. While most participants found measuring their blood pressure at home doable, many faced challenges. Participants’ experiences with five key factors influenced how easy or difficult their experience was: 1) Time, stress, and daily responsibilities; 2) Perceived importance of BP in pregnancy; 3) Role of family; 4) Capability of performing monitoring; 5) Convenience of monitoring.

**Conclusions:**

Among pregnant women in urban Ghana, home blood pressure monitoring was perceived as positive, important, and doable; however, challenges must be addressed.

## Background

Hypertensive diseases of pregnancy (HDP), including chronic hypertension, gestational hypertension, preeclampsia, and eclampsia, are important causes of adverse maternal and fetal outcomes globally [1, 2]. This burden is felt to a greater degree in low- and middle-income countries (LMICs), where both the incidence of HDP and the rate of poor outcomes is higher than in high-income countries (HICs) [3, 4]. This study was conducted in Ghana, where the incidence of HDP is estimated at 7.6%, and HDP is the most common direct cause of maternal death at many facilities, surpassing postpartum hemorrhage [5].

The increased incidence of HDP and risk of poor outcomes indicate a need for earlier diagnosis and intervention [6]. Barriers to early detection of HDP, such as low antenatal care attendance and long intervals between routine antenatal visits, are commonly faced in LMICs [7]. Home blood pressure monitoring (HBPM) is one possible approach to improve earlier detection of elevated BPs, and thus earlier diagnosis of HDP [8].

HBPM involves patients performing their own blood pressure (BP) measurements outside of a healthcare facility, and provides multiple measurements of a patient’s BP in their typical setting [9]. In high-income settings, HBPM has been shown to be accurate, consistent with readings in a usual clinical care setting, and accepted by women at higher risk of HDP [10–14]. Further, HBPM has been demonstrated to increase patient satisfaction and decrease stress [15]. However, investigation into the clinical benefit of HBPM relative to standard care has yielded mixed results in these settings [12, 15–18]. In LMICs, given the high risk for HDP complications and barriers to routine antenatal care, HBPM represents a unique opportunity for an impactful intervention. Integrating and maintaining HBPM during pregnancy may require additional support and training in LMIC settings where it is not commonplace [19]. This study aims to understand the experiences, facilitators, and barriers of HBPM for pregnant women in urban Ghana.

## Methods

### Setting

This study was conducted at Korle Bu Teaching Hospital (KBTH), the largest tertiary care facility in Accra, Ghana. KBTH provides antenatal care to both residents of Ghana’s urban capital city, as well as complex referral cases from throughout southern Ghana. The Department of Obstetrics and Gynaecology (OBGYN) staffs a six-floor maternity unit, providing antepartum, labor and delivery, and postpartum care, with 275 inpatient beds. HDP complicates 15% of the 10,000 annual deliveries at KBTH and has eclipsed postpartum hemorrhage as the leading cause of maternal mortality at the hospital [5].

### Participants

Participants were pregnant women who were receiving their antenatal care at KBTH between October 2022 and June 2023. Inclusion criteria were age ≥ 18 years, current pregnancy with gestational age ≤ 28 weeks, verbal fluency in English or local languages Twi or Ga, upper arm circumference appropriate for the BP cuff size (22–42 cm), and having a follow-up antenatal visit at KBTH either planned or scheduled. Exclusion criteria were admission to the hospital for inpatient care or need for emergent delivery or intervention.

### Recruitment

**Fig. 1:**
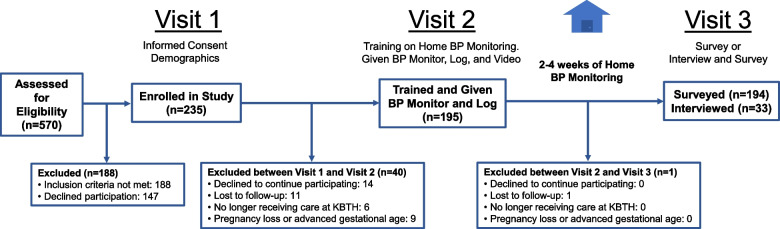
Study overview and enrollment flow diagram

Participants were recruited in the waiting room of the “booking clinic” at KBTH, where pregnant patients present for their first antenatal care visit. All pregnant women were screened; those meeting inclusion criteria underwent an informed consent process, and assenting participants were enrolled (Fig. 1). Written informed consent was obtained from all participants. Ethical approval was granted by the Institutional Review Board at KBTH (KBTH-IRB /0098/2021) and the University of Michigan (HUM00200589).

### Procedures

This was a concurrent triangulation mixed-methods study consisting of both surveys and semi-structured interviews. Study activities took place over three separate study visits, each coordinated with participants’ antenatal visits to KBTH (Fig. 1). Data collection was carried out by a team of three research assistants in participants’ choice of three languages (English, Twi, Ga). All research instruments were verbally administered. The day before the participant’s next visit was scheduled, research assistants communicated a reminder by telephone.

At the first study visit, participants were recruited and enrolled as detailed above. Information was collected on demographics, medical history, obstetric history, and prior experience with HBPM.

The second study visit was performed at the participant’s next antenatal visit. Participants were trained by a research assistant on the correct use of an automated BP monitor. Training consisted of a 5-min educational video in the participants’ language of choice (English, Twi, or Ga) and a hands-on interactive demonstration of the BP monitor. Participants were given an automatic BP monitor for home use and a paper log to record the date, time, and BP values, and were asked to return it at the completion of their involvement in this study. A Microlife WatchBP Home (Microlife AG Swiss Corporation, Switzerland) automated blood pressure monitor was used [20]. The model is intended for individual use and has been validated for use in pregnancy and pre-eclampsia [21]. This model was selected based on its relative affordability and availability in Accra. Participants were asked to monitor and log their BP once daily, consisting of two repeated BP measurements. If during their monitoring period they observed readings above 140 systolic or 90 diastolic, participants were told to report to the hospital or a local health care facility. This was communicated when the monitor was given to the participant at the second visit, both in the training video and verbally. We asked participants to bring their BP logs with them to their antenatal care visits and share them with their physicians so the BP data could be integrated into clinical decision making.

Participants were given contact information of the study coordinators and OBGYN physicians to contact if they were to have issues with the monitor.

The third study visit was scheduled after 2–4 weeks of home BP monitoring. Participants monitored at home for a mean of 28.6 days (SD 12.6). Surveys were administered to all participants and consisted of questions on five potential barriers to HBPM, as well as general attitudes toward HBPM for pregnant women in Ghana. Semi-structured interviews were conducted with a subset of participants, which expanded on these topics and also broadly addressed experiences with HBPM and attitudes towards HBPM. Interviews were initially conducted with every eligible participant. Once 50% of the anticipated number of interviews required to reach thematic saturation were conducted, additional interview participants were purposefully selected to ensure that the interview population reflected the demographic makeup of the overall study population with respect to age, highest level of education, parity, and diagnosis of chronic hypertension or HDP in a prior pregnancy. Interview transcription and review was conducted in an ongoing manner, and the final number of participants was determined by thematic saturation of data.

### Questionnaire development

Survey and interview questions were developed by the research team with the guidance of local obstetric healthcare providers. Questions were informed by prior research conducted by this team on anticipated facilitators of and barriers to HBPM from the perspective of obstetric providers at the study site [22]. Responses were assessed on a 4-point Likert scale, where 1 = Not at all; 2 = Not really, 3 = Mostly, and 4 = Definitely. All questions were piloted for understanding before use. Interview questions were designed to be open-ended and phrased in a way that did not lead the interviewee. This was done to avoid imposing the thoughts of researchers on the interviewee and to allow for responses that were not previously anticipated by the researcher.

### Analysis

Quantitative data analysis was performed using R version 4.2.2. Descriptive statistics were calculated for demographics and structured survey questions. Since this study was investigating a new concept and hypothesis testing was not the goal, we limited analysis to descriptive statistics. For the qualitative data, interviews were audio-recorded and transcribed verbatim. English interviews were transcribed using Descript 54.1.1, with subsequent manual review to ensure accuracy. Twi and Ga interviews were translated into English and transcribed using Microsoft Word, with bilingual researchers discussing and verifying any aspects of the translations that were not clear. A set of three transcripts were reviewed independently by two researchers, who developed initial codes. Next, they developed a codebook by reviewing successive sets of new transcripts in an iterative manner until key ideas were adequately captured. Finally, they uploaded the transcripts into Dedoose 9.0.78 for qualitative coding. The two researchers met regularly during the coding process to discuss and reconcile any new codes. Following the detailed coding process, common ideas were identified and consolidated into the major themes presented as the qualitative results below.

## Results

Of the 570 women who were screened for enrollment, 382 met inclusion criteria and 235 (61.5%) agreed to participate and were enrolled. Among the 235 enrolled participants, 194 (82.6%) completed the study (Fig. 1). Granular data was not collected on non-eligible participants, but given the high referral population to KBTH, the most common reason for ineligibility was advanced gestational age. Not all participants gave reasons for declining, but some were disinterest from the participant, non-approval from partner or other family member, or uncertainty about continuation of care at Korle Bu.

Of the 194 participants who were surveyed, 33 were also interviewed. Interviews were conducted in English (*N* = 19, 57.5%), Twi (*N* = 8, 24.2%), and Ga (*N* = 6, 18.1%). With respect to the greater study population, participants had a mean age of 31.6 (SD 5.3) years. 32.1% had not previously given birth (*N* = 62), 31.1% had less than a senior high school education (*N* = 60) (Table 1). Regarding prior experience with blood pressure, 9 (4.7%) had previously been diagnosed with a hypertensive disease of pregnancy, 3 of which were interviewed (9.1% of the interview population). 27 (14.3%) had previously monitored their own blood pressure, 2 of which were interviewed (6.3% of the interviewed population) (Table 2).

**Table 1:** Demographics

**Demographic Characteristic**	**All Participants (*N* = 194)**^**a**^	**Interview Participants (*N* = 33)**^**a**^
**Age, years**	31.6 (5.3)	31.2 (5.2)
**Gestational Age**	17w 3.7d (4w 4.9d)	17w 4.9d (5w 2.1d)
**Marital Status**		
Married	146 (77.3)	23 (71.9)
Not married^b^	43 (22.8)	9 (28.1)
**Highest Level of Education Completed**		
None	7 (3.6)	1 (3.0)
Primary school	7 (3.6)	2 (6.1)
Junior high school	46 (23.8)	10 (30.3)
Senior high school	56 (29.0)	7 (21.2)
Tertiary	77 (39.9)	13 (39.4)
**Monthly Household Income, Ghana cedis (USD)**^**c**^		
< 650 (< $56)	36 (36.4)	16 (53.3)
650–1000 ($56-$87)	32 (32.3)	8 (26.7)
1000–5000 ($87-$433)	27 (27.3)	6 (20.0)
5000–10000 ($433-$867)	2 (2.0)	0 (0.0)
> 10,000 (> $867)	2 (2.0)	0 (0.0)
**Insurance Status**		
No insurance	1 (0.5)	1 (3.0)
Public insurance (NHIS)	190 (98.5)	32 (97.0)
Private insurance	2 (1.0)	0 (0.0)
**Parity**	1.4 (1.5)	1.5 (1.4)
Nulliparous	62 (32.1)	10 (30.3)
Multiparous	131 (67.9)	23 (69.7)
**Current Pregnancy Gestation Number**		
Singleton	171 (93.4)	30 (90.9)
Twins	12 (6.6)	3 (9.1)

Data presented as Mean (SD) for continuous variables and n (%) for categorical variables

*NHIS* National Health Insurance Scheme

^a^Categories may not total to overall *N* values due to missingness

^b^Includes cohabitating, single, divorced, widowed

^c^Ghana Cedi to US Dollar conversion based on 0.087 conversation rate on June 24, 2023

**Table 2:** Previous experience with clinical blood pressure disease or monitoring

**Experience Factor**	**All Participants** **(*N* = 194)**^**a**^	**Interview Participants (*N* = 33)**^**a**^
**Hypertensive Disorder in Prior Pregnancy**		
No	184 (95.3)	30 (90.9)
Yes	9 (4.7)	3 (9.1)
**Chronic Hypertension**		
No	179 (92.8)	28 (84.9)
Yes	14 (7.3)	5 (15.2)
**Family History of Preeclampsia**^b^		
No	169 (88.0)	28 (87.5)
Yes	23 (12.0)	4 (12.5)
**Prior Experience Measuring Own BP**		
No	162 (85.7)	30 (93.8)
Yes	27 (14.3)	2 (6.3)

Data presented as n (%)

*BP* Blood pressure

^a^Categories may not total to overall *N* values due to missingness

^b^Mother or sister

### Overall attitudes towards HBPM

Overwhelmingly, participants reported liking the process and experience of measuring their blood pressure at home.

*“Yes, I loved to do it at home. The truth is I was so happy when I got the machine, because BP in pregnancy is something very scary. So, when I had it, I was so happy I could check my BP at home. I enjoyed checking my BP at home.”*

- 36-year-old woman, P2, with a junior high school education

The main reason participants liked HBPM was that the ability to have knowledge about the health of their pregnancy was important to them.

*“I just wanted to know my BP. Whether I’m in good condition with my baby, where everything is. [If my] blood pressure is low or high. I wanted to know that.”*

- 24-year-old woman, P0, with a senior high school education

Some specifically liked knowing their BP because it would allow them to take action accordingly, like resting or seeking medical care.

*“It’s good, because if you are working, you don’t know whether [your BP] is low or it’s high. but with the machine, if you wake up in the morning, you can check and then you know how to do things.”*

- 30-year-old woman, P1, with a junior high school education

*“I am happy when I use it… I wanted to check [my BP] at home because if my BP goes high or low, I would see it and then I can rush to the hospital.”*

- 29-year-old woman, P1, with a junior high school education

### Factors influencing how easy or hard HBPM was for participants

Most women found HBPM easy to perform. Of those who did face challenges, many were able to overcome those challenges and monitor their BP daily. However, others missed days of monitoring due to the challenges.

*“It’s not a difficult task. Once you wake up in the morning, you can easily do it and get on with your daily activities.”*

- 29-year-old woman, P3, with a primary school education

*“In the beginning, it was difficult because I haven’t used the machine before. However, it became easy over time and I was able to do it exactly as it is. It became easier overtime because I didn’t give up and I kept doing it.”*

- 20-year-old woman, P0, with a tertiary education

**Fig. 2:**
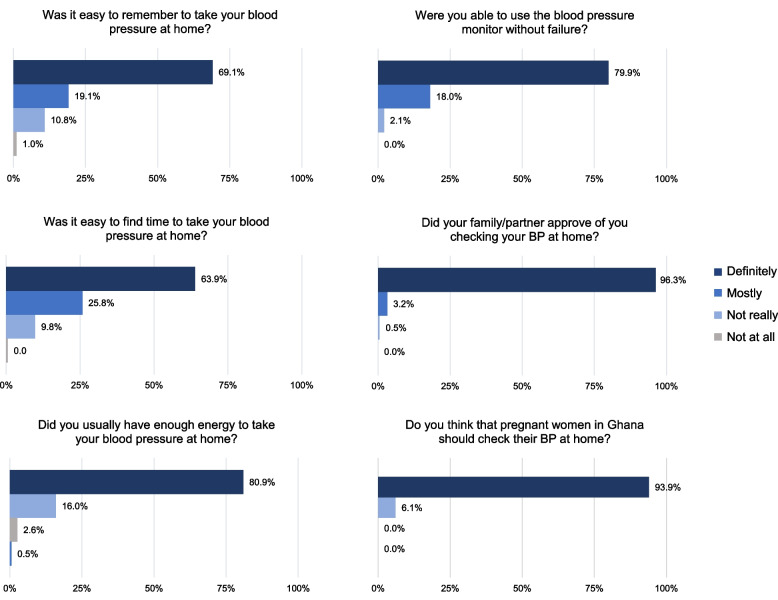
Factors impacting the experience of home blood pressure monitoring (*N* = 194)

**Fig. 3:**
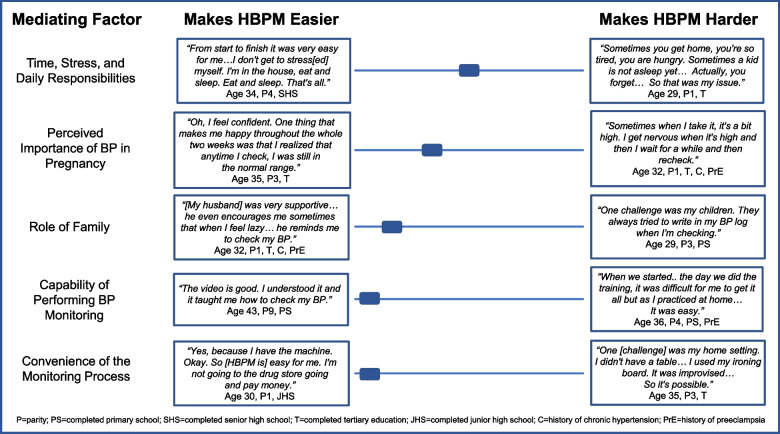
Relative impact of each factor on making home blood pressure monitoring easier or harder. The slide bar indicates the overall assessment of each factor making HBPM easier or harder, based on the volume and strength of qualitative comments and integration of quantitative data

Quantitative and qualitative data converged to demonstrate that participants’ experiences with five key factors influenced how easy or hard HBPM was for them: 1) Time, stress, and daily responsibilities; 2) Perceived importance of BP in pregnancy; 3) Role of family; 4); Capability of performing BP monitoring; 5) Convenience of the monitoring process (Figs. 2 and 3).

#### Time, stress, and daily responsibilities

Almost all participants referenced responsibilities to their families or their professions as factors impacting their ability to perform HBPM. The comments primarily highlighted stress or lack of time.

*“The challenge I will have is my routines… As a woman, I have to take care of the house, going to work, taking care of the kids. So sometimes I do forget. That’s the only thing I think is challenging… because I have to make sure the kids go to school. And after that you are at work. So if you don’t make it a conscious effort, you can’t do it.”*

- 35-year-old woman, P3, with a tertiary education

Of these participants, about half were able to overcome these challenges and monitor every day. The other half explained that they missed days or compromised their process due to lack of time or stress from daily activities. This was supported by survey data, which showed that 63.9% of participants felt it was easy to find time to check their BP and 80.9% had enough energy to check their BP (Fig. 2).

*“I leave early in the morning [so, sometimes] I did it earlier before leaving… If the car comes earlier and I am not done… [then] when I get back home, then I do it.”*

- 35-year-old woman, P0, with a tertiary education and chronic hypertension

*“Sometimes on some days when I have to go to work or I’m busy, I am not able to check.”*

- 29-year-old woman, P2, with a junior high school education

Conversely, a few participants reported having surplus free time or very little stress. These participants unanimously explained that this made it easy for them to monitor their BP every day.

*“I was able to check from that day we did the training. I even checked this morning… I’m not doing anything at home. So sometimes, I feel like doing something, so I just check.”*

- 30-year-old woman, P1, with a senior high school education

Several participants also reported difficulty remembering to take their BP. They primarily cited work and family responsibilities occupying their minds as the reason they forgot. Survey data supported these results, with 11.9% of participants reporting they had difficulty remembering to take their BP daily (Fig. 2).

*“What made it difficult for me, and then I missed some of the days I was supposed to check, was personal activities. Coming from work late or maybe too much work, and then I forget to take or record my BP.”*

- 33-year-old woman, P0, with a tertiary education

#### Perceived importance of BP in pregnancy

Many participants explained that knowing the importance of having elevated BP in pregnancy made monitoring easier because it drove their motivation to measure consistently. This was supported by survey data, which demonstrated that 95.9% of respondents felt that pregnant women in Ghana should be monitoring their BP daily (Fig. 2).

*“I told myself I have to do this for myself and my baby. So if this hypertension thing will put my baby at risk and checking every day will help come out of that risk, I have to.”*

- 35-year-old woman, P0, with a tertiary education and chronic hypertension

Related to the perceived importance of BP in pregnancy, nearly all participants referenced having an emotional response, either positive or negative, to their BP values. The majority of participants had low or normal BP values, and their emotional response was always positive. Feelings of reassurance and emotional comfort were most commonly expressed.

*“[My normal BP] made me feel… that I’m not in a bad condition. So I feel okay. I feel good.”*

- 32-year-old woman, P2, with a senior high school education

This was especially relevant in situations where participants were worried that busy daily activities, including household chores, caring for other children, and working outside the home, could negatively impact their health in pregnancy.

“*The children got me so angry. All over the place. So I have to sleep for a while and check [my BP]. But when I checked it, everything was normal. I was okay with it.”*

- 34-year-old woman, P4, with a senior high school education

Fewer participants experienced high BP values. Among them, most described being frightened or nervous in response to the reading. While most continued measuring despite recording high values, one participant explained that they did not want to re-check their BP following an elevated reading.

*“I don’t like checking it…especially when I check and the figure is high... then I’ll start thinking about it. What exactly did I do? I’m taking my medicine. So what is going on wrong? To be frank, I don’t really like checking. But for a day, that one day I will check and know what is going on. But after I check and it’s high, I just want to pause.”*

- 32-year-old woman, P1, with a tertiary education and history of chronic hypertension and preeclampsia who has previously monitored her blood pressure at home

This participant later explained that they would monitor their blood pressure at home daily during pregnancy and while breast feeding if asked to do so by their doctor, explaining that their fear is specifically associated with rechecking following high values.

*“***Participant:*** I would love to check. I would love to check, but not after checking… I don’t mind checking it every day.*

**Interviewer: ***So, if a doctor asks you to check your blood pressure every day of your 9 month pregnancy?*

**Participant: ***I’ll check.*

**Interviewer: ***And breastfeeding, period too?*

**Participant: ***I’ll check.”*

Many, but not all participants reported they would panic if they got a high reading despite never seeing one. None of those participants said fear of seeing a high value would deter them from checking. Some people specifically said that it’s important to know their BP value, even if it is frightening to check.

*“It made me feel good. I liked to confirm I was okay. I wanted to know if my bp was low or high. Because it’ll be [more] helpful to know if it’s high than to be frightened to check.”*

- 36-year-old woman, P4, with a primary school education and a history of preeclampsia

#### Role of family

Most participants referenced family making their experience easier in some capacity. Comments referred primarily to approval and help from family, which ranged from reminders to check their BP to assistance arranging the cuff properly on their arm. Survey data showed that 96.3% of participants had the approval of their family to monitor their BP at home (Fig. 2).

*“My husband was supportive, because sometimes even when he’s at work, he can call to find out if I’ve checked. It sounds like a joke but he’s serious. Sometimes when he comes home from work in the evening he asks if I’ve checked. If I say no, he brings the machine for me… Sometimes he’s the one who [applies] the machine for us to do it”*

- 30-year-old woman, P1, with a senior high school education

While most family-related factors were explained as exclusively helpful by participants, one family-related challenge was disruptions from younger children.

*“Sometimes the kids. You have to wait when they’re not around… They would do something that you would talk. That was the only challenge… Aside from that everything was cool.”*

- 33-year-old woman, P2, with a tertiary education

#### Capability of performing BP monitoring

Another important factor that impacted participants’ ease with HBPM was their capability and comfort with the monitoring process.

*“The truth is, anytime I am checking my BP I do not think what I’m doing is wrong or a mistake. I know what I am doing is correct, because I have confidence in what I am doing, because I believe I am doing the right thing. I am not afraid.”*

- 27-year-old woman, P1, with a junior high school education

Most participants explained that receiving training on use of the monitor prior to engaging in HBPM was important in building their ability to monitor at home. These qualitative responses were consistent with survey data demonstrating that 79.9% of respondents reported no technical challenges when using the BP monitor (Fig. 2).

*“I didn’t go to school but when I did [the training], it showed me a lot of things. It made me know that it’s not only those who went to school that can do this.”*

- 33-year-old woman, P4, who has previously monitored her blood pressure at home

Most participants said using the BP monitor was very easy, while a few reported difficulties Common difficulties were getting the cuff into the proper location on the arm, discomfort when the cuff inflated, and the machine reporting an "error." Most people who had difficulties with the cuff said that it got easier as time went on, and that they were able to overcome that challenge either by themselves or with help.

*“In the beginning, it was difficult to tie the cuff and align the artery mark properly but my mum helped me with it… it became easier over time.”*

- 20-year-old woman, P0, with a tertiary education

#### Convenience of the monitoring process

Finally, many participants also reported that they enjoyed the convenience of performing HPBM and gaining knowledge about their BPs at home. They explained that HBPM was preferred over their other option for BP monitoring—to have it performed at a local pharmacy—because it saved them time and cost.

*“Yes [I liked HBPM] because it helps me to know my BP at home. I don’t have to go to the pharmacy and pay someone to check for me.”*

- 40-year-old woman, P3, with a junior high school education

The majority of participants had a setting at home conducive to HBPM, including a place to sit with their back supported and a place to rest their arm.

*“At home, there’s a chair and a table. So I was relaxed. After doing my household chores, I just take my chair and relax then I check my BP.”*

- 36-year-old woman, P4, with a primary school education and a history of preeclampsia

The contrary was true for a few participants, which presented a challenge to following the correct positioning to perform HBPM. Some were unable to follow proper technique to support their backs or arms while measuring their BP, while others found makeshift solutions in their home.

*“In the bedroom there’s no chair, so I just sit on the bed… You ask me to sit somewhere [where] my shoulder should be relaxing, but sometimes I just sit and then check. It’s difficult to find a comfortable position.”*

- 32-year-old woman, P1, with a tertiary education and a history of chronic hypertension and preeclampsia

For a summary graphic demonstrating each of the five factor’s relative impact on making HBPM easier or harder, see Fig. 3.

## Discussion

Overall, pregnant women in our sample overwhelmingly reported that they liked monitoring their BP at home. The primary facilitator for HBPM was a desire to gain knowledge about their health, which was driven by the perceived importance of BP in pregnancy. The most common barrier to HBPM was time, stress, and daily responsibilities, which resulted in participants not having time to monitor or forgetting to monitor. Participants’ experiences with HBPM were also influenced by their level of family support, which contributed to their success. Pregnant women reported high capability with the monitoring process after a training session, practice, or help from family, which supported their ability to engage in HPBM. Finally, the convenience of the monitoring process was a facilitator for the majority of participants in saved time, travel, and cost, and most had a suitable home setting for HBPM. Quantitative and qualitative results converged across each measured factor. Of note, one qualitative finding was not anticipated and thus was not assessed in survey questions: the convenience of home monitoring, which emerged as a key facilitator.

A body of research has been conducted to understand the facilitators and barriers to HBPM in hypertensive patients, mostly conducted in non-pregnant hypertensive patients and in HICs [23–27]. In agreement with this study, availability of time, difficulty incorporating HBPM into daily schedules, and difficulty remembering have previously been identified as primary barriers to consistent HBPM [23, 27]. These studies have also identified knowledge of HBPM protocol, concerns over cuff accuracy, difficulty using the cuff, and thinking that office readings were sufficient were barriers to HBPM. These were not barriers that our participants reported facing, likely due to the standardized training they received. This finding, along with other studies demonstrating low pre-training capability in HBPM, highlight the importance of HBPM education and training before a monitoring period [25]. Multiple studies have cited the cost of the monitor to the patient as a potential barrier to HBPM [28, 29]. This was not a factor in our study since monitors were provided free of charge; however, this has been cited by obstetric providers at our study site as a potential barrier and is likely to be a notable barrier in LMICs [22].

Previous research has found that participants believed the benefits of HBPM to outweigh barriers, which is consistent with our findings that participants had a high perceived importance of HBPM [24, 28]. Importantly, the level of understanding of the importance of HBPM has been linked to adherence [29]. In addition, participants’ confidence in HBPM was increased if HBPM was recommended by their doctor, supporting the need for healthcare provider buy-in [22]. Perceived healthcare autonomy has been associated with improved patient-perceived competence and satisfaction [30, 31]. This agrees with our findings that participants liked HBPM because it gave them involvement in and knowledge of their health. It has been demonstrated that clinical outcomes can be improved by involving and empowering patients in their care across contexts [12, 30–32]. Studies linking HBPM to improvements in blood pressure control have yielded mixed results, with some studies showing lower blood pressures in women engaged in home monitoring [17], while other studies showing no significant difference [12, 18]. Of note, all of these studies looking at clinical outcomes have been conducted in high income settings.

This study demonstrated that the majority of participants liked engaging in HBPM, were able to overcome challenges to successfully monitor at home, and found the convenience of having the monitor at home to be a significant facilitator to regular BP monitoring. If integrated into clinical practice, HBPM in pregnant women represents an avenue for possible early detection of HDP. This may be of particular benefit in Ghana and other LMICs where barriers exist to consistent antenatal care. More research is required to quantify the increased frequency of BP monitoring among pregnant women engaged in HBPM compared to standard antenatal care, and to understand the impact that HBPM may have on clinical outcomes in LMIC settings. Demonstrated clinical impact could justify the expansion of HBPM programs in urban Ghana and the provision of governmental or external funding for automated BP monitors.

This study identified aspects of home monitoring that were helpful and challenging. These key elements should be addressed with protocol adjustments and patient education in future attempts to integrate HBPM into antenatal care in this and similar settings. Time, stress, and daily responsibilities were the most commonly reported barrier to daily monitoring. Thus, clinical monitoring protocols should balance the benefits of frequent monitoring data with the importance of not over-burdening patients. Potential strategies to overcome this barrier include the use of cellphone technology to send patients daily reminder alerts. Of note, despite the magnitude of participants’ reporting schedule as a barrier, HBPM is likely to lead to more frequent BP checks than in standard antenatal care.

Participants’ perceived importance of BP in pregnancy was a key facilitator of HBPM, motivating patients to monitor their BP regularly. This highlights the importance of patient education prior to engaging in HBPM to emphasize the role and impact of BP in pregnancy. This was also linked to the observed high positive and negative emotionality in response to BP values, which was unanticipated and not otherwise reported in the literature. In addition to technical HBPM training, education is needed to balance patients’ appreciation for the importance and potential severity of elevated BP, without promoting fear as a deterrent to checking BP. Anxiety around checking BP was not specifically assessed but we identified it as an area of future research.

The role of family as a facilitator of HBPM participation represents an area of possible innovation. Especially for participants with low health literacy or numeracy, family involvement in HBPM training and execution may allow this intervention to be successful in pregnant women at highest socioeconomic risk. The unanticipated yet strong role of family support in this study can be considered a factor that may increase participation in similar health maintenance interventions in similar settings. Family members’ willingness to check their own blood pressure unprompted also represents an interesting opportunity to reach more patients with health maintenance interventions. More research is needed to understand how family can be helpful for this and other health interventions in Ghana and other LMICs.

### Strengths and limitations

A major strength of this study was the implementation and assessment of an at-home monitoring experience in a key and under-studied LMIC pregnant population. A mixed methods design facilitated an in-depth exploration of barriers and facilitators of HBPM in a real-world setting. Limitations of this study include its focus at a single large urban hospital, meaning results may not be applicable outside of this setting. While the use of English, Twi, and Ga in training and research activities included the majority of patients at the hospital, a few spoke other local languages and were not able to be included. However, in seeking a highly representative perspective in this context, the study did include participants with a wide range of education, income levels, and parity. Importantly, the home monitoring period in this study was limited to 2–4 weeks. Further research is necessary to understand if this approach is effective through the duration of a pregnancy and what different facilitators and barriers may be present during a longer period of home monitoring.

Since this is a novel topic in low resource settings, we intentionally defined our study population broadly to gather perspectives from all pregnant women. We did not limit recruitment to women with a prior or current history of an HDP because HDP are very common in Ghanaian pregnant populations and HDP can arise in pregnancies without any risk factors. As a result, most participants in our study had not previously been diagnosed with chronic hypertension or HDP. This meant that the perspective of participants with hypertension or previous HDP, who may benefit most from HBPM, became the perspective of the minority within our study population. This is a limitation of our study, but it is notable that of the participants with chronic hypertension or previous HDP, all but one said they enjoyed checking their BP because it was important to be able to seek care when necessary. Next steps for this research include focusing on highest risk populations and extending monitoring through the entire pregnancy.

## Conclusions

Overall, pregnant women in Ghana like checking their blood pressure at home, see it as doable, and believe that HBPM should supplement routine antenatal care. Participants’ experiences with five major factors influenced how easy or hard HBPM was for them. These factors should be considered when designing future research and clinical care structured around HBPM in Ghana and other LMICs. The integration of HBPM into antenatal care for pregnant women in Ghana and other LMICs represents an avenue for improvement not only in patients’ engagement in their own care, but also potentially in clinical outcomes.

## Data Availability

The dataset generated and/or analyzed during the current study are not publicly available due to the data (qualitative interview transcripts) containing specific, personal, and detailed information about the participants, but are available from the corresponding author on reasonable request. The important and representative information is available in the quotations and tables in the manuscript.

## Abbreviations

BP: Blood pressure
HBPM: Home blood pressure monitoring
HDP: Hypertensive disorders of pregnancy
HICs: High-income countries
KBTH: Korle Bu Teaching Hospital
LMICs: Low- and middle-income countries
OBGYN: Obstetrics and Gynaecology
